# Author Correction: Molecular correlates of cisplatin-based chemotherapy response in muscle invasive bladder cancer by integrated multi-omics analysis

**DOI:** 10.1038/s41467-022-29627-4

**Published:** 2022-04-04

**Authors:** Ann Taber, Emil Christensen, Philippe Lamy, Iver Nordentoft, Frederik Prip, Sia Viborg Lindskrog, Karin Birkenkamp-Demtröder, Trine Line Hauge Okholm, Michael Knudsen, Jakob Skou Pedersen, Torben Steiniche, Mads Agerbæk, Jørgen Bjerggaard Jensen, Lars Dyrskjøt

**Affiliations:** 1 Department of Molecular Medicine, Aarhus University Hospital, Aarhus, Denmark; 2 Department of Clinical Medicine, Aarhus University, Aarhus, Denmark; 3 Department of Pathology, Aarhus University Hospital, Aarhus, Denmark; 4 Department of Oncology, Aarhus University Hospital, Aarhus, Denmark; 5 Department of Urology, Aarhus University Hospital, Aarhus, Denmark

**Keywords:** Cancer, Molecular biology, Biomarkers, Medical research, Molecular medicine

Correction to: *Nature Communications* 10.1038/s41467-020-18640-0, published online 25 September 2020.

The original version of this Article omitted patient survival information from the Source Data and Supplementary Source Data files. In the Source Data file a new column ‘Deceased’ has been added to the data for Fig. 2 (Column J), Fig. 4 (Column C) and Fig. 6 (Column F). In the Supplementary Source Data file a new column ‘Deceased’ has been added to Supplementary Table 2 (Column H) and Supplementary Table 3 (Column F). The Source Data files have been updated.

## Supplementary information

Updated Supplementary Source Data

Updated Source Data

