# Supplementary material for: Molecular correlates of cisplatin-based chemotherapy response in muscle invasive bladder cancer by integrated multi-omics analysis
Source: Nat Commun. 2020 Sep 25;11:4858. doi: 10.1038/s41467-020-18640-0 (PMC7519650; doi:10.1038/s41467-020-18640-0)
Supplement: Supplementary file 1 — Supplementary Information [file 41467_2020_18640_MOESM1_ESM.pdf]

Supplementary Information for

Molecular Correlates of Cisplatin-based Chemotherapy Response in Muscle Invasive Bladder Cancer by Integrated Multi-omics Analysis

Taber, Christensen and Lamy *et al.*

Supplementary information

**Supplementary figures**	**2**
**Supplementary tables**	**10**
**Overview of reagents, software tools and data sets**	**14**
**Supplementary references**	**19**

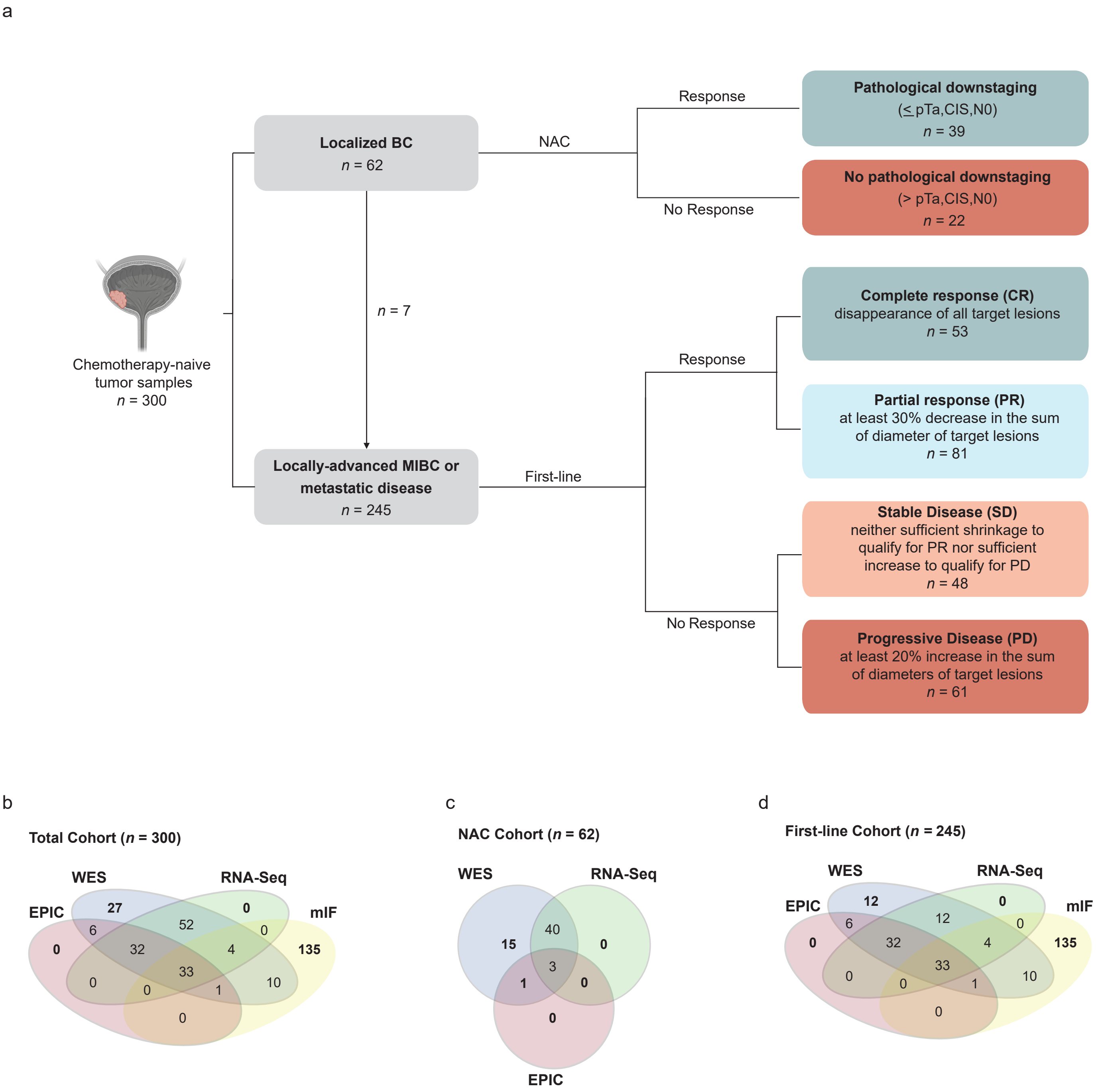

Supplementary Figure 1. Overview of 300 patients with bladder cancer (BC) included in the study. a) Patients with localized BC (T1-T4a, N0, M0) were treated with cisplatin-based neoadjuvant chemotherapy (NAC) followed by radical cystectomy (CX). Treatment response was evaluat-ed based on the pathological examination of the CX specimen. NAC treatment response was not available for one patient. Recurrence after CX was observed, and seven patients received first-line treatment for metastatic disease. First-line treatment response was based on pre-(baseline) and post-treatment PET/CT or MRI, CT and X-ray examination. Representative metastatic sites of all involved organs, were identified as target lesions at the Dep. of Radiology, AUH. Treatment response was evaluated using RECIST 1.1 response criteria. First-line treatment response was not availible for two patients. Image created with BioRender.com. b-d) Venn diagram illustrating the overlap between the platforms used for molecular analysis: whole exome sequencing (WES), Illumina EPIC 800k methylation array (EPIC), QuantSeq 3´mRNA sequencing (QuantSeq), multiplex immunofluorescence (mIF). Source data are provided as a Supplementary Source Data file.

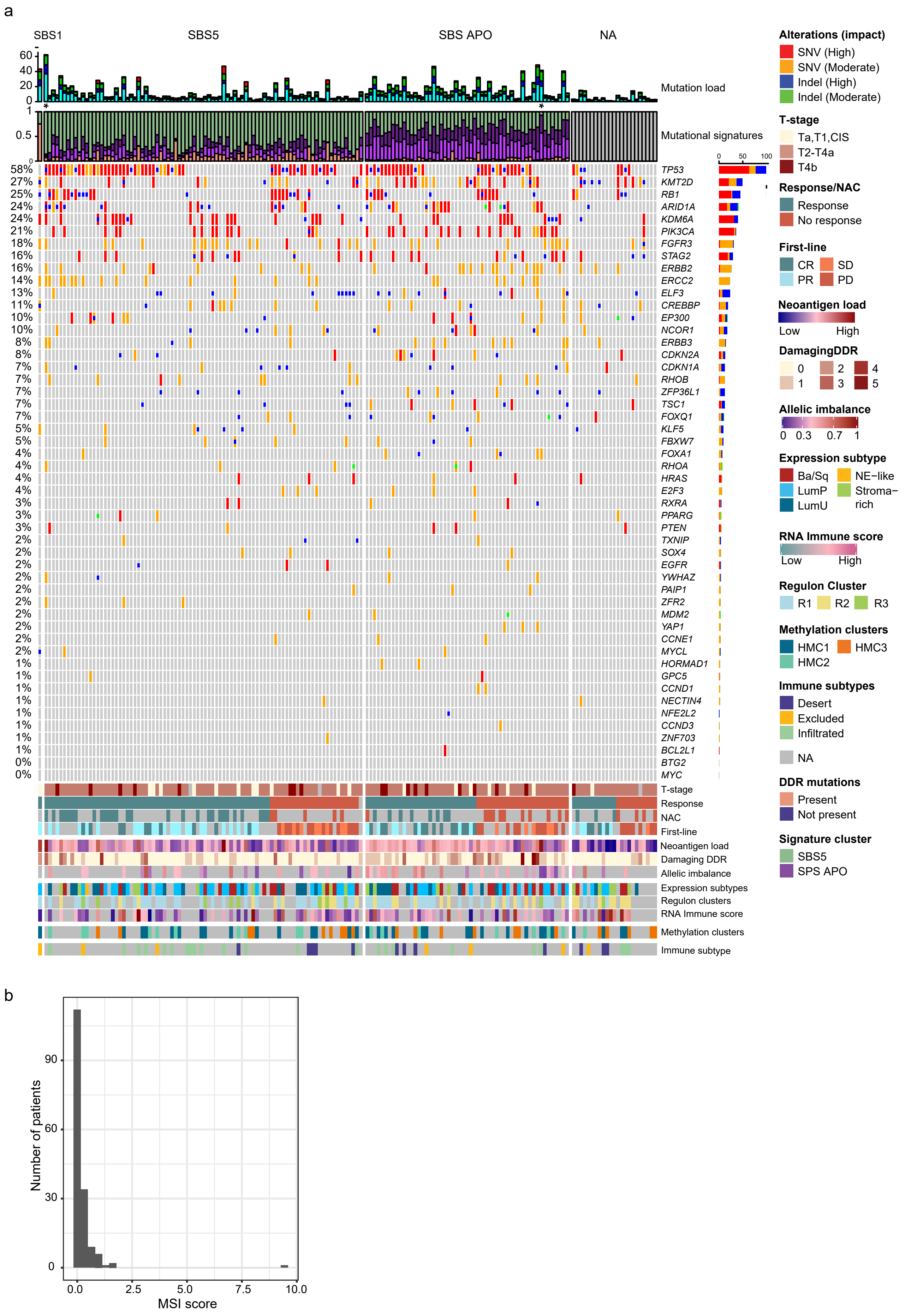

Supplementary Figure 2. Overview of the genomic alterations correlated to chemotherapy response and MSI status. a)Oncoplot showing the significantly mutated genes or copy-number affected genes from Robertson et al. (TCGA) in 165 tumors annotated by exome coverage, mutation load stratified by impact (as defined by SnpEff) and mutational signature deconvolution (top panels) and by clinical response, number of damaging mutations in DDR genes, percentage of genome in allelic imbalance, expression subtypes, regulon cluster, RNA immune score, hypermethylation cluster and immune phenotype (bottom panel). Samples are sorted as in Figure 1. b) Distribution of MSI score derived from MSIsensor for all patients. Source data are provided as a Supplementary Source Data file.

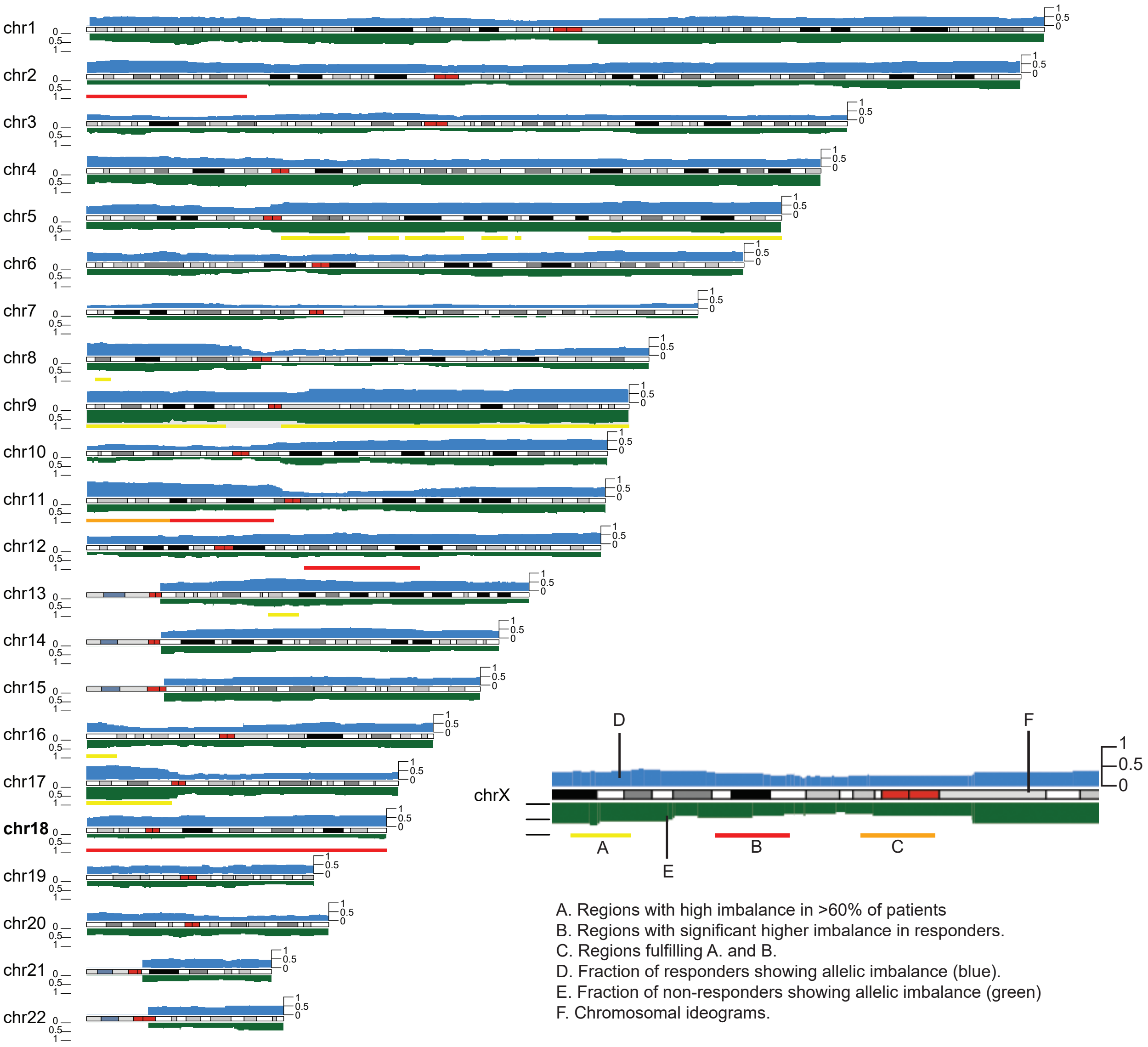

Supplementary Figure 3. Genomic landscape of allelic imbalance in relation to response status. For each chromosome, the fraction of patients showing allelic imbalance is shown in blue above the ideograms for patients responding to treatment and in green below the ideograms for patients non-responding to treatment. Genomic regions under allelic imbalance in more than 60% of the patients are marked in yellow. Genomic regions that have significantly higher allelic imbalance in responders versus non-responders are marked in red. Genomic regions marked in orange fulfill both conditions. Source data are provided as a Supplementary Source Data file.

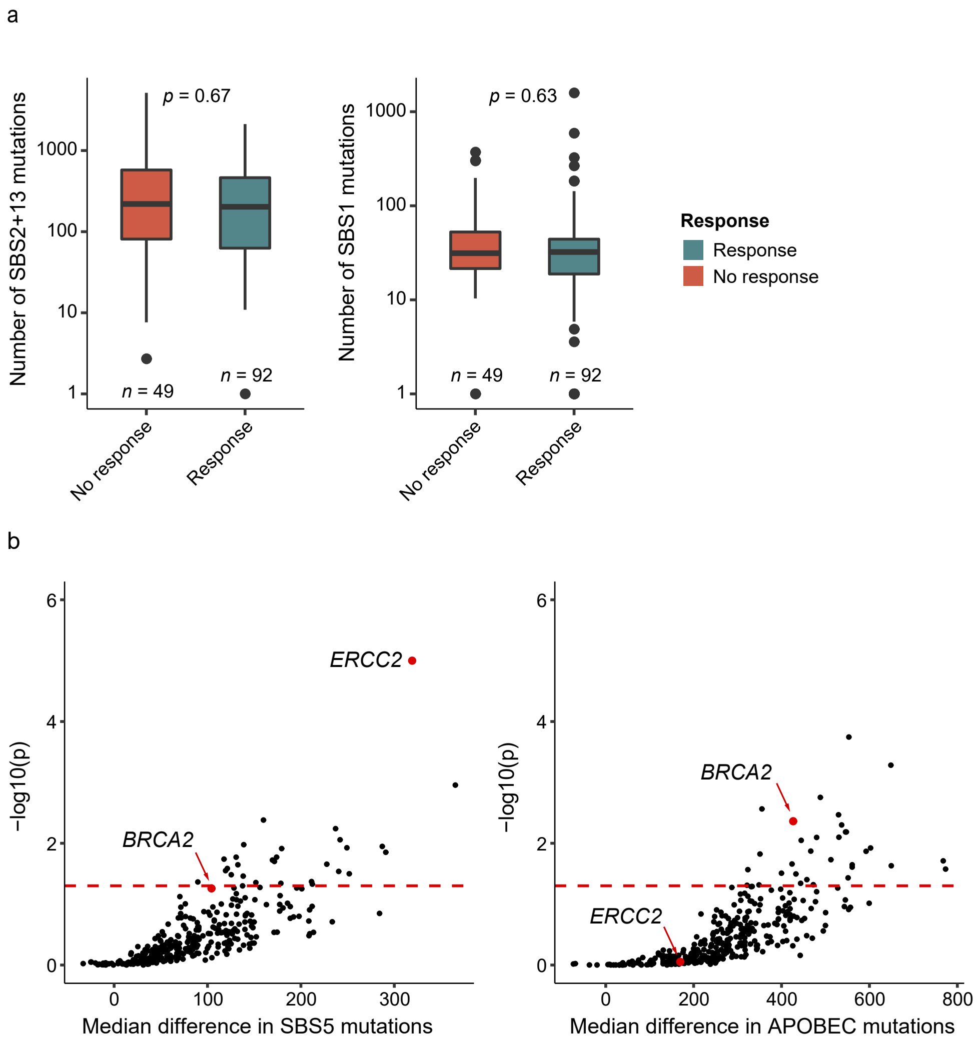

Supplementary Figure 4. Signature specific mutations in relation to response and gene mutation status.
a) Number of SBS2+13 (APOBEC) mutations (left) and number of SBS1 mutations (right) in relation to chemotherapy response. P-values were calculated using a Wilcoxon rank sum test. For all boxplots, the center line represents the median, box hinges represent first and third quartiles, whiskers represent ±1.5 x interquartile range (IQR) and points represent outliers. b) Volcano plots showing the difference between the median number of mutations for mutated tumors and the median number of mutations for wild-type tumors for all genes mutated in more than 5% of TCGA data (only mutations with moderate- or high protein impact are considered). The left panel represents the number of mutations in an SBS5 context and the right panel represents the number of mutations in an SBS2+13 (APOBEC) context. P-values were calculated using a permutation test (n=100,000) that controls for mutation burden per sample and gene. The red dashed lines indicate significance levels at p = 0.05. Source data are provided as a Supplementary Source Data file.

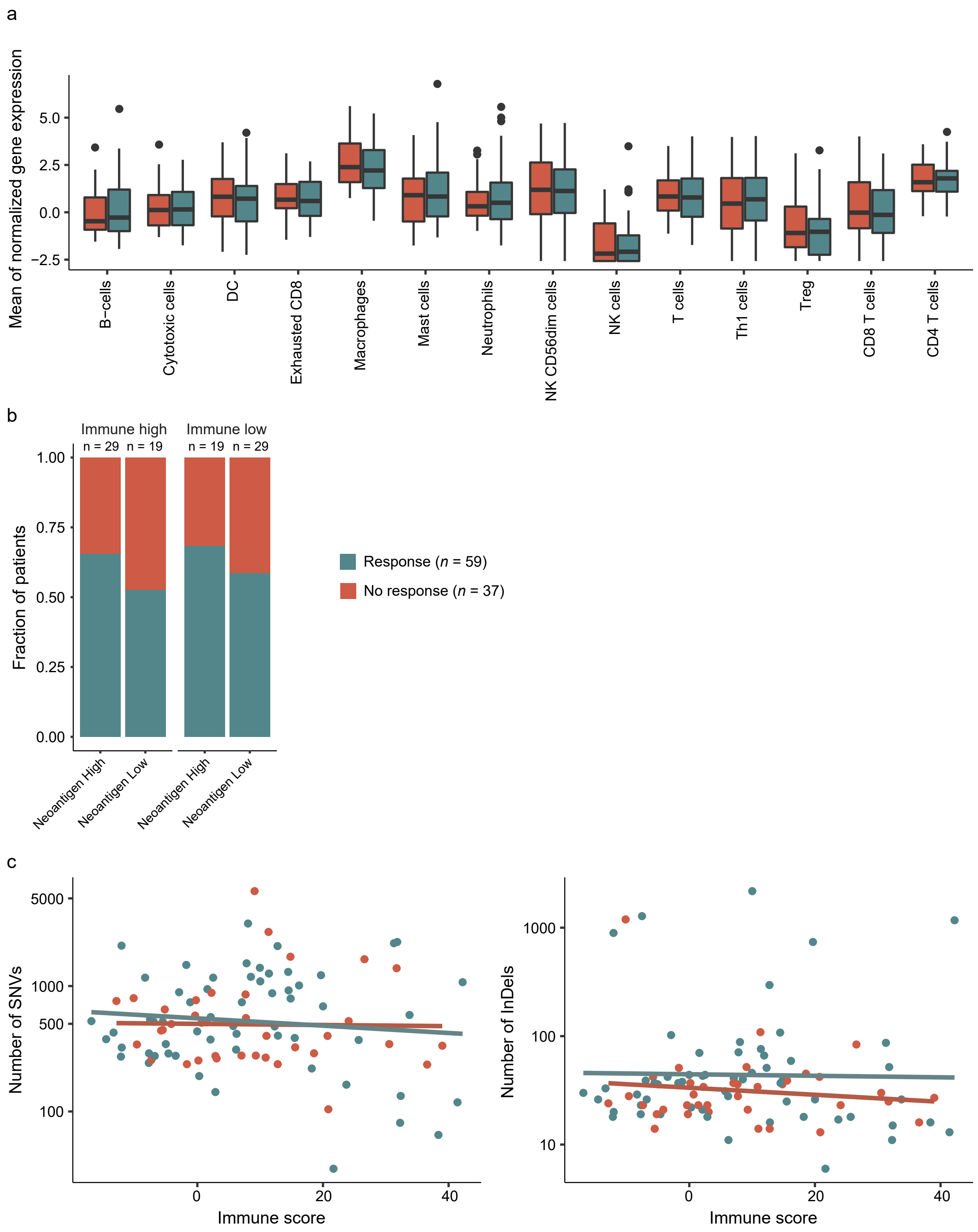

Supplementary Figure 5. Immune infiltration in relation to chemotherapy response, SNVs and InDels
a) Estimated immune cell levels based on RNA-seq data in relation to chemotherapy response. For all boxplots, the center line represents the median, box hinges represent first and third quartiles, whiskers represent ±1.5 x interquartile range (IQR) and points represent outliers.
b) Dichotomization of patients based on neoantigen load and RNA-seq based immune score and relation to chemotherapy response.
c) RNA-seq based immune score in relation to SNVs (left) and InDels (right) and stratified by chemotherapy response. Source data are provided as a Supplementary Source Data file.

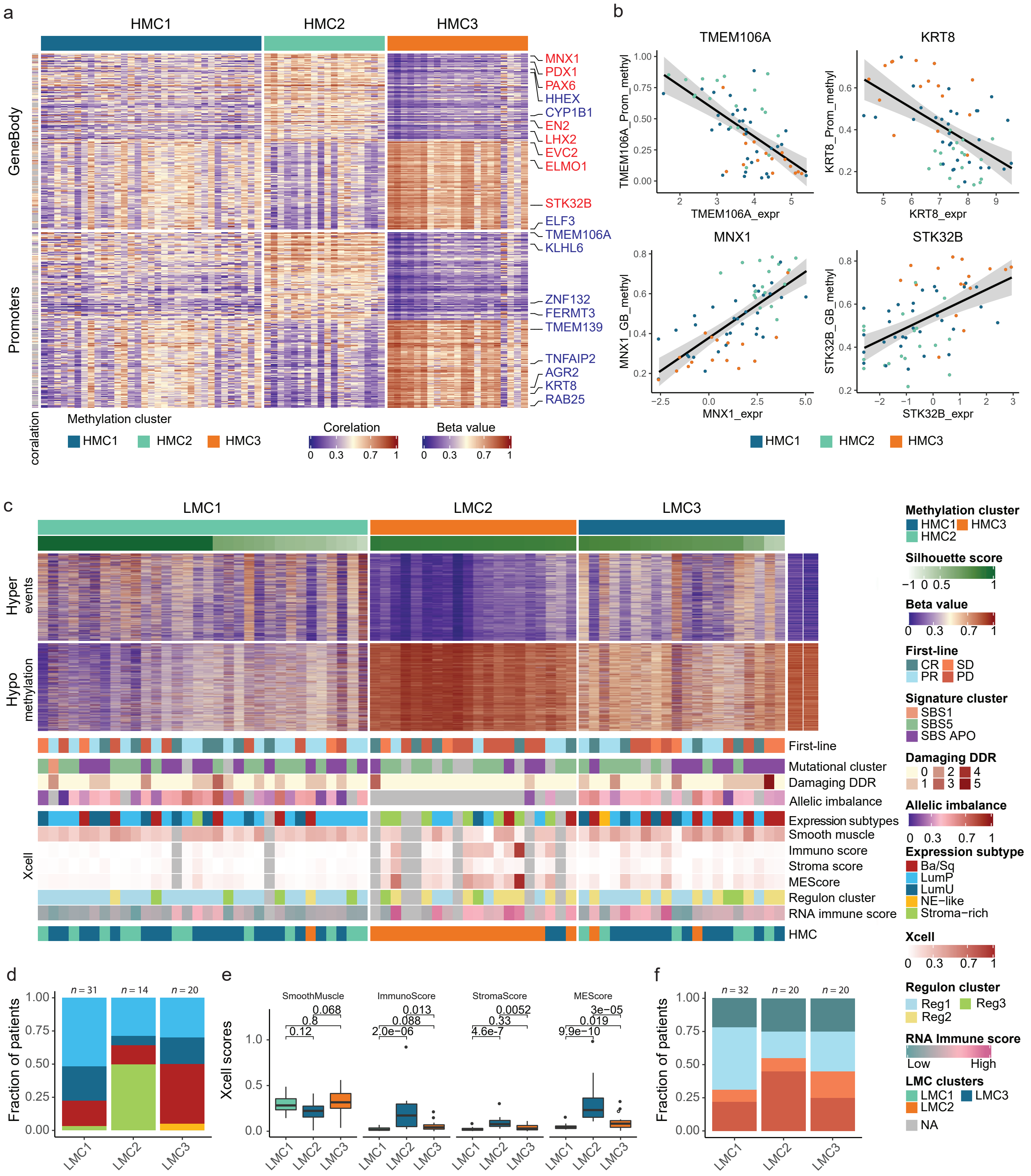

Supplementary Figure 6. Integration of the hypermethylation clusters with gene expression and definition of the clusters based on the hypomethylated cancer-specific CpG sites. a) Integration of significant promoter or gene body methylation pattern and corresponding gene expression. CpGs have been summarised to obtain a single methylation measurement for promoter or gene body for all genes. The top 200 significant genes between the two extreme clusters defined in Figure 4, HMC2 and HMC3, (100 with high methylation in HMC2, 100 with high methylation in HMC3) are presented here. The correlation with gene expression is shown on the left of the heatmap with red color showing positive correlation and blue color showing negative correlation. Gene names with an absolute correlation to expression above 0.5 are marked on the right side with font color showing the direction of the correlation (red for positive and blue for negative correlation). b) Four examples of gene expression vs promoter methylation (top) or gene body methylation (bottom). Samples are represented by a dot colored as the HMC cluster they belong to. C-F) DNA methylation subtypes based on hypomethylated cancer-specific CpG sites. The light-grey font represents the 95% confidence interval for the smoothed mean calculated using a linear regression model. c) Clustering of samples based on hypomethylation events (n=5000). Heatmap shows beta values and the right panel shows normal bladder and leukocyte beta values for comparison. d) Methylation clusters compared to gene expression subtypes. e) Gene set scores calculated using XCell and stratified by methylation clusters. (LMC1: n = 30; LMC2: n = 14; LMC3: n = 20) f) RECIST response measurements stratified by methylation clusters. MEscore = Microenvironment score. P-values were calculated using a Wilcoxon rank sum test. For all boxplots, the center line represents the median, box hinges represent first and third quartiles, whiskers represent ±1.5 x interquartile range (IQR) and points represent outliers. Source data are provided as a Supplementary Source Data file.

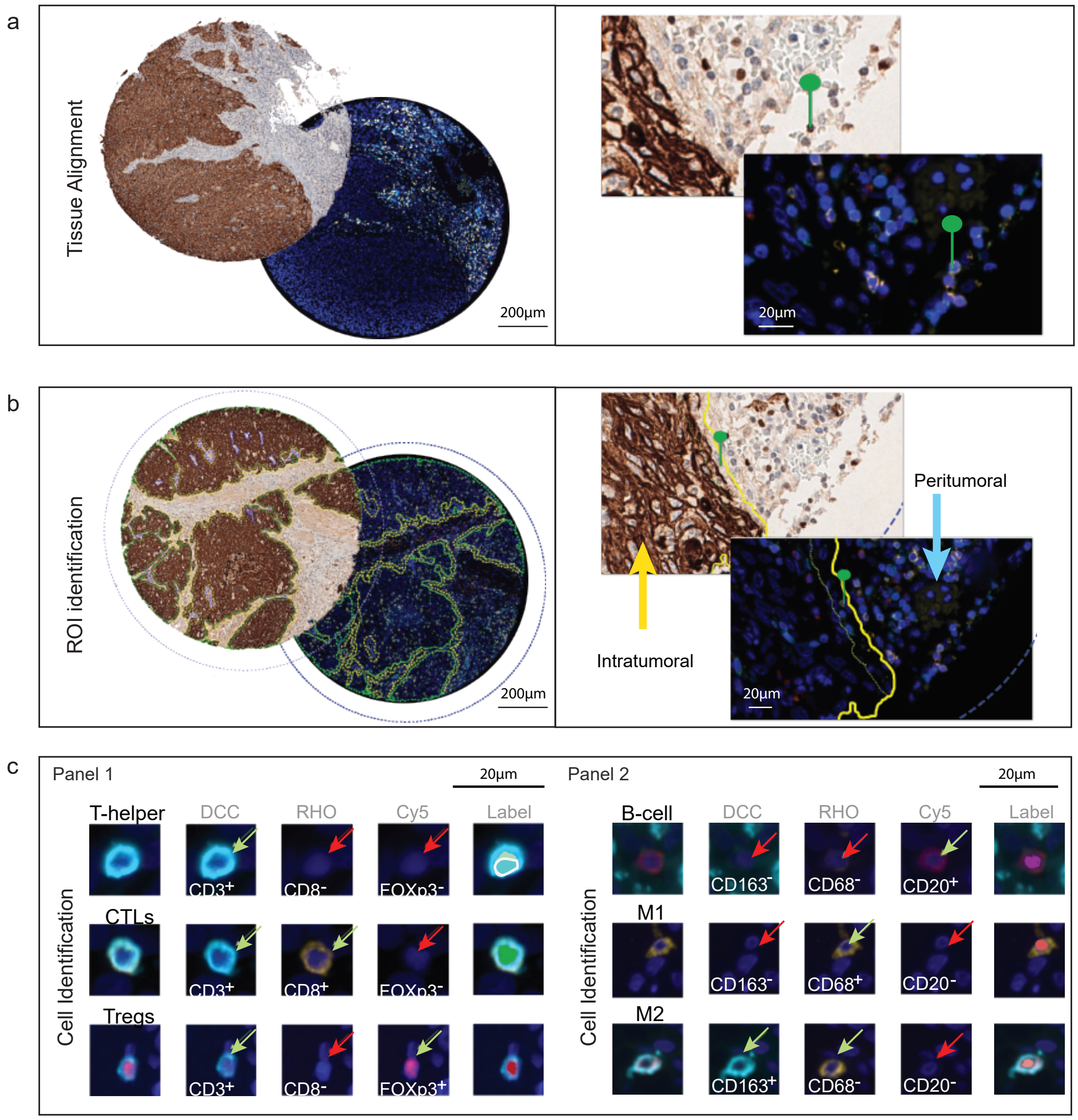

Supplementary Figure 7. Immunostaining performed on bladder cancer tissue microarray samples from 184 patients. All protein measurements were performed once for each distinct sample. Representative images illustrating the multiplex image analysis protocol. a) Alignment of the immunohistochemistry (IHC) and immunofluorescence (IF) staining results using the Visiopharm Tissuealign™ module, illustrated with a tissue core (right) and a section (left). Green marks indicates the precise cell-to-cell alignment between the two staining results. b) The cytokreatin staining is used to define the region of interest (ROI), the ROI is then transferred to the IF layer. Cells located in the tumor parenchyma are defined as intratumoral (yellow arrow), and cells located in the stroma surrounding the tumor parenchyma are defined as peritumoral cells (blue arrow). c) Classification of immune cells based on co-localization of selected markers for panel 1 and 2. Green arrows indicate positive IF-staining and red arrows negative IF-staining.

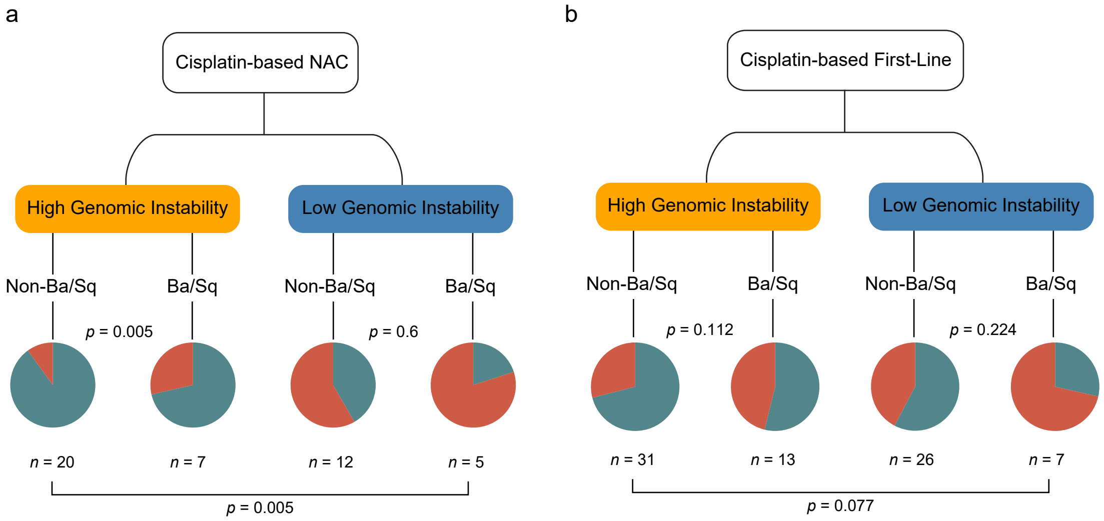

Supplementary Figure 8. Integrative analysis for patients treated with NAC and First-line separately. Integration of genomic and transcriptomic data for patients treated with a) NAC and b) First-Line displaying likelihood of cisplatin-based chemotherapy response. P-values were calculated using a Fisher's exact test. Source data are provided as a Supplementary Source Data file.

Supplementary Table 1. Clinical characteristics and multi-omics platforms

	Total (*n*=300)	Genomics (*n*=165)	Transcriptomic (*n*=121)	Epigenetics (*n*=72)	Proteomics (*n*=183)
**Age at diagnosis**					
Mean ± SD, y	64 ± 8	64 ± 8	64 ± 8	64 ± 8	64 ± 7
Range	41– 86	41 - 80	41 - 80	41-77	44 - 86
**Follow up time**					
Mean ± SD, m	38 ± 37	34 ± 31	31 ± 27	37 ± 33	43 ± 42
Range	4 - 217	4 - 175	4 - 175	6- 175	5 - 208
**Sex**					
Female	65 (21.7%)	38 (23.0%)	28 (23.1%)	19 (26.4%)	37 (20.2%)
Male	235 (78.3%)	127 (77.0%)	93 (76.9%)	53 (73.6%)	146 (79.8%)
**Smoking**					
Non-smoker	67 (22.3%)	29 (17.6%)	25 (20.7%)	15 (20.8%)	45 (24.6%)
Smoker	215 (71.7%)	124 (75.2%)	89 (73.6%)	50 (69.4%)	128 (69.9%)
Unknown	18 (6.0%)	12 (7.3%)	7 (5.8%)	7 (9.7%)	10 (5.5%)
**T stage at diagnosis**					
Ta,T1,CIS	26 (8.7%)	19 (11.5%)	14 (11.6%)	9 (12.5%)	13 (7.1%)
T2-T4a	228 (76.0%)	126 (76.4%)	93 (76.9%)	52 (72.2%)	138 (75.4%)
T4b	43 (14.3%)	18 (10.9%)	13 (10.7%)	11 (15.3%)	30 (16.4%)
Unknown	3 (1.0%)	2 (1.2%)	1 (0.8%)	0 (0.0%)	2 (1.1%)
**N stage at diagnosis**					
N0	167 (55.7%)	109 (66.1%)	77 (63.6%)	38 (52.8%)	82 (44.8%)
N1	39 (13.0%)	22 (13.3%)	15 (12.4%)	9 (12.5%)	23 (12.6%)
N2	68 (22.7%)	23 (13.9%)	21 (17.4%)	17 (23.6%)	56 (30.6%)
N3	6 (2.0%)	3 (1.8%)	3 (3.5%)	2 (2.8%)	4 (2.2%)
Unknown	20 (6.7%)	7 (4.2%)	5 (4.1%)	6 (8.3%)	18 (9.8%)
**M stage at diagnosis**					
M0	235 (78.3%)	137 (83.0%)	97 (80.2%)	52 (72.2%)	133 (72.7%)
M+	53 (17.7%)	22 (13.3%)	20 (16.5%)	15 (20.8%)	42 (23.0%)
Unknown	12 (4.0%)	6 (3.6%)	4 (3.3%)	5 (9.6%)	8 (4.4%)
**Treatment***					
NAC	62 (20.6%)	55 (33.3%)	44 (36.7%)	4 (5.5%)	3 (1.6%)
First-Line	245 (81.7%)	110 (66.7%)	81 (64.8%)	72 (100%)	183 (100%)
**Response**					
No response	125 (41.7%)	60 (36.4%)	43 (35.5%)	30 (41.7%)	81 (44.3%)
Response	172 (57.3%)	104 (63.0%)	78 (64.5%)	42 (58.3%)	100 (54.6%)
Unknown	3 (1.0%)	1 (0.6%)	0	0	2 (1.1%)
**NAC response***					
No response	22 (35.5%)	20 (33.3%)	15 (34%)	3 (75%)	2 (66%)
Response	39 (62.9%)	39 (65%)	29 (65.9%)	1 (25%)	1 (33%)
Unknown	1 (1.6%)	1 (1.6%)	0	0	0
**First-line response**					
No response	109 (44.5%)	44 (40%)	31 (38.3%)	30 (41.7%)	81 (44.3%)
Response	134 (54.7%)	66 (60%)	50 (61-7%)	42 (58.3%)	100 (54.6%)
Unknown	2 (1.2%)	0	0	0	2 (1.1%)

* NAC response was defined as pathological downstating to $\leq$CIS, Ta or T1 based on the pathological examination on the cystectomy specimen. First-line treatment response was defined as Complete or Partial response (RECIST v 1.1.) based on pre- (baseline) and post-treatment PET/CT or MRI, CT and x-ray examination. Source data are provided as a Supplementary Source Data file.

Supplementary Table 2. Clinical Characteristics and treatment regimes (NAC vs First-line)

	NAC (*n*=62)*	First-Line (*n*=245)*
**Age at diagnosis**		
Mean ± SD, years	64 ± 8	64 ± 8
Range	42 - 76	41- 86
**Follow up time**		
Mean ± SD, months	29 ± 11	40 ± 41
Range	5 - 60	4 - 217
**Sex**		
Female	11 (17.7%)	57 (23.3%)
Male	51 (82.3%)	188 (76.7%)
**Smoke**		
Non-smoker	10 (16.1%)	57 (23.3%)
Smoker	52 (83.9%)	170 (69.4%)
Unknown	0	18 (7.3%)
**T stage at diagnosis**		
Ta,T1,CIS	2 (3.2%)	24 (9.8%)
T2-T4a	60 (96.8%)	175 (71.4%)
T4b	0	43 (17.6%)
Unknown	0	3 (1.2%)
**N stage at diagnosis**		
N0	61 (98.4%)	113 (46.1%)
N1	1 (1.6%)	38 (15.5%)
N2	0	68 (27.8%)
N3	0	6 (2.4%)
Unknown	0	20 (8.2%)
**M stage at diagnosis**		
M0	62 (100%)	181 (73.9%)
M+	0	53 (21.6%)
Unknown	0	11 (4.5%)
**Treatment Regimes**		
GC	61 (98.4%)	203 (82.9%)
**GCx3 + Gencitabinx3	0	1 (0.4%)
**GCx2 + Gemcitabinx3	0	1 (0.4%)
**GCx1 + Gemcitabinx5	0	1 (0.4%)
MVAC	0	23 (9.4%)
GCT	0	9 (3,7%)
Cisplatin+Etoposide	0	1 (0.4%)
Gemcitabin	0	5 (2.0%)
Carboplatin+Etoposide	1 (1.6%)	0
Carboplatin+Gemcitabin	0	1 (0.4%)
**Completed Series**		
1	1 (1.6%)	0
2	8 (12.9%)	0
3	8 (12.9%)	29 (11.8%)
4	45 (72.6%)	22 (9.0%)
5	0	13 (5.3%)
≥6	0	181 (73.9%)
**Response*** **		
No response	22 (35.5%)	109 (44.5%)
Response	39 (62.9%)	134 (54.7%)
Unknown	1 (1.6%)	3 (1.2%)

* Seven patients had both neoadjuvant chemotherapy (NAC) and first-line treatment.
** Three patients had a change in treatment regime during treatment. xN indicates the number of completed series.
*** NAC response was defined as pathological downstating to ≤CIS, Ta or T1 based on the pathological examination on the cystectomy specimen. First-line treatment response was defined as Complete or Partial response (RECIST v 1.1.) based on pre- (baseline) and post-treatment PET/CT or MRI, CT and x-ray examination.Source data are provided as a Supplementary Source Data file.

Supplementary Table 3. Selected genes involved in DNA damage response pathways

Gene name	DNA damage response pathway
MLH1	Mismatch repair
MSH2	Mismatch repair
MSH6	Mismatch repair
PMS1	Mismatch repair
PMS2	Mismatch repair
ERCC2	Nucleotide excision repair
ERCC3	Nucleotide excision repair
ERCC4	Nucleotide excision repair
ERCC5	Nucleotide excision repair
BRCA1	Homologous recombination
MRE11A	Homologous recombination
NBN	Homologous recombination
RAD50	Homologous recombination
RAD51	Homologous recombination
RAD51B	Homologous recombination
RAD51D	Homologous recombination
RAD52	Homologous recombination
RAD54L	Homologous recombination
BRCA2	Fanconi anemia
BRIP1	Fanconi anemia
FANCA	Fanconi anemia
FANCC	Fanconi anemia
PALB2	Fanconi anemia
RAD51C	Fanconi anemia
BLM	Fanconi anemia
ATM	Key regulator of DDR
ATR	Key regulator of DDR
CHEK1	Cell cycle control
CHEK2	Cell cycle control
MDC1	Cell cycle control
POLE	Other
MUTYH	Other
PARP1	Other
RECQL4	Other

Supplementary Table 4. Bladder cancer associated transcription factors used for regulon analysis

ESR1
ESR2
AR
PGR
PPARG
RARA
RARB
RARG
RXRA
RXRB
RXRG
ERBB2
ERBB3
FGFR1
FGFR3
FOXA1
FOXM1
GATA3
GATA6
HIF1A
KLF4
STAT3
TP63

Transcription factors were obtained from[1].

Overview of reagents, software tools and data sets

REAGENT or RESOURCE	SOURCE	IDENTIFIER
**Antibodies**		
Anti-CD8 [C8/144B], dilution: 1:150	Dako, Agilent	cat#M710301-2
Anti-CD3 [2GV6], Ready to use	Ventana Medical Systems, Inc.	cat#790-4341
Anti-FOXP3 [SP97], dilution 1:10, RRID: AB_2537884	Thermo Fisher	cat#MA5-16365
Anti-CD163 [MRQ-26], Ready to use	Ventana Medical Systems, Inc.	cat#760-4437
Anti-CD68 PF-M1 [PG-M19], dilution 1:100	Dako	cat#GA61361-2
Anti-CD20 [L26], Ready to use	Ventana Medical Systems, Inc.	cat#760-2531
Anti-HLA class 1 ABC antibody [EMRB-5], dilution 1:100	Abcam	cat#ab70328
PD-L1 [Sp263], Ready to use, RRID:AB_2819099	Ventana Medical Systems, Inc.	cat#790-4905
PD-1 [NAT105], Ready to use	Ventana Medical Systems, Inc.	cat#760-4895
Pan Cytokeratin [AE1/3], dilution 1:100	Dako	cat#GA005361-2
anti-rabit-HRP (GaR-HRP), Ready to use, OmniMap anti-Rb HRP (RUO), DISCOVERY	Ventana Medical Systems, Inc.	cat#760-4311
anti-mouse-HRP (GaM-HRP), Ready to use, OmniMap anti-Ms HRP (RUO), DISCOVERY	Ventana Medical Systems, Inc.	cat#760-4310
**Biological Samples**		
Fresh Frozen tissue specimens, FFPE tissue specimens and Tissue Microarrays (TMA)	Department of Urology, Aarhus University Hospital	N/A
**Chemicals, Peptides, and Recombinant Proteins**		
DISC Inhibitor	Ventana Medical Systems, Inc.	cat#760-4840
UltraView Universal 3,3’-Diaminobenzidin (DAB)	Ventana Medical Systems, Inc.	cat#760-500
Anti-fade mounting medium with DAPI	VECTAshield	cat#H-1200

carboxyrhodamine-6G-Tyramide (Ty-R6G), RTU	Ventana Medical Systems, Inc.	cat #760-244
FAM (Carboxyfluorescein)-Tyramide (Ty-FAM), Ready to use	Ventana Medical Systems, Inc.	cat #760-243
diethylaminocoumarin-tyramide (Ty-DCC), Ready to use	Ventana Medical Systems, Inc.	cat #760-240
sulphoCy5-tyramide (Ty-Cy5), Ready to use	Ventana Medical Systems, Inc.	cat #760-238
EZ Prep solution	Ventana Medical Systems, Inc.	cat #950–102
Hematoxylin II	Ventana Medical Systems, Inc.	cat#790-2208
Bluing reagent	Ventana Medical Systems, Inc.	cat#760-2037
Reaction Buffer	Ventana Medical Systems, Inc.	cat#950-300
LCS (Liquid coverslip)	Ventana Medical Systems, Inc.	cat#650-210
CC1 (High pH buffer)	Ventana Medical Systems, Inc.	cat#950-124
CC2 (low pH buffer)	Ventana Medical Systems, Inc.	cat#950-223
**Critical Commercial Assays**		
KAPA Hypr Prep 96/24 Library kit	Roche	K8504/KK8502
Twist Human Core Exome EF Multiplex Complete Kit	TWIST Bioscience	PN 1000803
Infinium Methylation EPIC Kit	Illumina	WG-317-1003
3‘ mRNA-Seq Library Prep Kit FWD HT	LEXOGEN	015.1x96
**Deposited Data**		
WES data	This paper	EGAS00001004507
Expression data	This paper	EGAS00001004505
Copy number data	This paper	EGAS00001004519
Methylation data	This paper	EGAS00001004515
Normalized gene expression data	This paper	Data file 4
WES and methylation TCGA data	[1]	https://portal.gdc.cancer.gov/

Leukocyte methylation data (450k)	2	http://www.ncbi.nlm.nih.gov/geo/query/acc.cgi?token=pjszvekkmmaeyzu&acc=GSE32148
**Software and Algorithms**		
R version 3.6.1	The R project for statistical Computing	https://www.r-project.org/
GATK version 3.7	Genome Analysis Toolkit	https://gatk.broadinstitute. org/
VarScan2 version 2.4.1	3	http://dkoboldt.github.io/varscan/
Bam-readcount v0.7.4	NA	https://github.com/genome/bam-readcount
PolyPhen-2	4	http://genetics.bwh.harvard.edu/pph2/
MutationAssessor v3	5	http://mutationassessor.or g/r3/
SnpEff v4.3i	6	http://snpeff.sourceforge.n et/
SomaticSignatures v2.24.0	7	http://bioconductor.org/packages/release/bioc/html/SomaticSignatures.html
MutationalPatterns v2.0.0	8	https://bioconductor.org/packages/release/bioc/html/MutationalPatterns.html
RTN v2.12.0	9	https://bioconductor.org/packages/release/bioc/html/RTN.html
xCell (Web tool)	10	https://xcell.ucsf.edu/
GenomeStudio v2.0.4	Illumina	https://support.illumina.com/array/array_software/genomestudio/downloads.ht ml
ASCAT v2.3	11	https://github.com/Crick-CancerGenomics/ascat
ChAMP v2.8.6	12	http://bioconductor.org/packages/release/bioc/html/ChAMP.html

ConsensusClusteringPlus1.48.0	13	http://bioconductor.org/packages/release/bioc/html/ConsensusClusterPlus.html
Salmon v0.10.0	14	https://github.com/COMBINE-lab/salmon
tximport v1.12.3	15	https://bioconductor.org/packages/release/bioc/html/tximport.html
edgeR v.3.26.8	16	https://bioconductor.org/packages/release/bioc/html/edgeR.html
consensusMIBC v1.1	17	https://github.com/cit-bioinfo/consensusMIBC
Visiopharm version 2018.9.5.5952: Visiopharm Tissue Array module, Visiopharm Tissue Align module, Visiopharm Tissue Author module	Visiopharm	https://www.visiopharm.com/module
REDCap 9.1.8	18	https://www.project-redcap.org/
bcl2fastq2 v2.17	Illumina	https://support.illumina.com/
bwa_mem v 0.7.5	19	http://bio-bwa.sourceforge.net/
Polysolver v1.0	20	https://software.broadinstitute.org/cancer/cga/polysolver
Trim Galore! v0.4.1	NA	https://www.bioinformatics.babraham.ac.uk/projects/trim_galore/
Picard suite v2.7.1	NA	https://broadinstitute.github.io/picard/
samtools suite v1.6.0	21	http://samtools.sourceforge.net/
MuTect2 (GATK v3.7)	22	https://github.com/broadinstitute/gatk
survminer 0.4.7	NA	https://github.com/kassambara/survminer
survival 3.1-12	NA	https://github.com/therneau/survival
**Other**		
Hamamatsu NanoZoomer s60 Digital Slide Scanner	Meyers Instruments	-

Hamamatsu Nanozoomer 2.0 HT	Meyers Instruments	-
COSMIC mutational signatures v3	-	https://cancer.sanger.ac.uk/cosmic/signatures

Supplementary references

1. Robertson, A. G. *et al.* Comprehensive Molecular Characterization of Muscle-Invasive Bladder Cancer. *Cell* **171**, 540–556.e25 (2017).
2. Harris, R. A. *et al.* Genome-wide peripheral blood leukocyte DNA methylation microarrays identified a single association with inflammatory bowel diseases. *Inflamm. Bowel Dis.* **18**, 2334–2341 (2012).
3. Koboldt, D. C. *et al.* VarScan 2: somatic mutation and copy number alteration discovery in cancer by exome sequencing. *Genome Res.* **22**, 568–576 (2012).
4. Adzhubei, I. A. *et al.* A method and server for predicting damaging missense mutations. *Nat. Methods* **7**, 248–249 (2010).
5. Reva, B., Antipin, Y. & Sander, C. Predicting the functional impact of protein mutations: application to cancer genomics. *Nucleic Acids Res.* **39**, e118 (2011).
6. Cingolani, P. *et al.* A program for annotating and predicting the effects of single nucleotide polymorphisms, SnpEff: SNPs in the genome of Drosophila melanogaster strain w1118; iso-2; iso-3. *Fly* **6**, 80–92 (2012).
7. Gehring, J. S., Fischer, B., Lawrence, M. & Huber, W. SomaticSignatures: inferring mutational signatures from single-nucleotide variants. *Bioinformatics* **31**, 3673–3675 (2015).
8. Blokzijl, F., Janssen, R., van Boxtel, R. & Cuppen, E. MutationalPatterns: comprehensive genome-wide analysis of mutational processes. *Genome Med.* **10**, 33 (2018).
9. Castro, M. A. A. *et al.* Regulators of genetic risk of breast cancer identified by integrative network analysis. *Nat. Genet.* **48**, 12–21 (2016).
10. Aran, D., Hu, Z. & Butte, A. J. xCell: digitally portraying the tissue cellular heterogeneity landscape. *Genome Biol.* **18**, 220 (2017).
11. Van Loo, P. *et al.* Allele-specific copy number analysis of tumors. *Proc. Natl. Acad. Sci. U. S. A.* **107**, 16910–16915 (2010).
12. Tian, Y. *et al.* ChAMP: updated methylation analysis pipeline for Illumina BeadChips. *Bioinformatics* **33**, 3982–3984 (2017).
13. Wilkerson, M. D. & Neil Hayes, D. ConsensusClusterPlus: a class discovery tool with confidence assessments and item tracking. *Bioinformatics* vol. 26 1572–1573 (2010).
14. Patro, R., Duggal, G., Love, M. I., Irizarry, R. A. & Kingsford, C. Salmon provides fast and bias-aware quantification of transcript expression. *Nat. Methods* **14**, 417–419 (2017).
15. Soneson, C., Love, M. I. & Robinson, M. D. Differential analyses for RNA-seq: transcript-level estimates improve gene-level inferences. *F1000Res.* **4**, 1521 (2015).

16. Robinson, M. D., McCarthy, D. J. & Smyth, G. K. edgeR: a Bioconductor package for differential expression analysis of digital gene expression data. *Bioinformatics* vol. 26 139–140 (2010).
17. Kamoun, A. *et al.* A Consensus Molecular Classification of Muscle-invasive Bladder Cancer. *Eur. Urol.* (2019) doi:10.1016/j.eururo.2019.09.006.
18. Harris, P. A. *et al.* The REDCap consortium: Building an international community of software platform partners. *J. Biomed. Inform.* **95**, 103208 (2019).
19. Li, H. & Durbin, R. Fast and accurate short read alignment with Burrows–Wheeler transform. *Bioinformatics* **25**, 1754–1760 (2009).
20. Shukla, S. A. *et al.* Comprehensive analysis of cancer-associated somatic mutations in class I HLA genes. *Nat. Biotechnol.* **33**, 1152–1158 (2015).
21. Li, H. *et al.* The Sequence Alignment/Map format and SAMtools. *Bioinformatics* **25**, 2078–2079 (2009).
22. Gonzalez-Perez, A. *et al.* IntOGen-mutations identifies cancer drivers across tumor types. *Nature Methods* vol. 10 1081–1082 (2013).
