# Supplementary material for: Molecular correlates of cisplatin-based chemotherapy response in muscle invasive bladder cancer by integrated multi-omics analysis
Source: Nat Commun. 2020 Sep 25;11:4858. doi: 10.1038/s41467-020-18640-0 (PMC7519650; doi:10.1038/s41467-020-18640-0)
Supplement: Supplementary file 3 — Description of Additional Supplementary Files [file 41467_2020_18640_MOESM3_ESM.pdf]

**Description of Additional Supplementary Files**

File Name: Supplementary Data 1
Description: Clinicopathological and treatment data

File Name: Supplementary Data 2
Description: Antibody panels, dilutions and reagents

File Name: Supplementary Data 3
Description: Whole exome sequencing metrics

File Name: Supplementary Data 4
Description: Normalized mRNA read counts
