# Supplementary material for: Molecular correlates of cisplatin-based chemotherapy response in muscle invasive bladder cancer by integrated multi-omics analysis
Source: Nat Commun. 2020 Sep 25;11:4858. doi: 10.1038/s41467-020-18640-0 (PMC7519650; doi:10.1038/s41467-020-18640-0)
Supplement: Supplementary file 8 — Reporting Summary [file 41467_2020_18640_MOESM8_ESM.pdf]

nature research

Corresponding author(s): Prof. Lars Dyrskjøt

Last updated by author(s): Aug 21, 2020

Reporting Summary

Nature Research wishes to improve the reproducibility of the work that we publish. This form provides structure for consistency and transparency in reporting. For further information on Nature Research policies, see our Editorial Policies and the Editorial Policy Checklist.

Statistics

For all statistical analyses, confirm that the following items are present in the figure legend, table legend, main text, or Methods section.

n/a	Confirmed	
☐	☒	The exact sample size (*n*) for each experimental group/condition, given as a discrete number and unit of measurement
☐	☒	A statement on whether measurements were taken from distinct samples or whether the same sample was measured repeatedly
☐	☒	The statistical test(s) used AND whether they are one- or two-sided<br>*Only common tests should be described solely by name; describe more complex techniques in the Methods section.*
☐	☒	A description of all covariates tested
☐	☒	A description of any assumptions or corrections, such as tests of normality and adjustment for multiple comparisons
☐	☒	A full description of the statistical parameters including central tendency (e.g. means) or other basic estimates (e.g. regression coefficient) AND variation (e.g. standard deviation) or associated estimates of uncertainty (e.g. confidence intervals)
☐	☒	For null hypothesis testing, the test statistic (e.g. *F*, *t*, *r*) with confidence intervals, effect sizes, degrees of freedom and *P* value noted<br>*Give P values as exact values whenever suitable.*
☒	☐	For Bayesian analysis, information on the choice of priors and Markov chain Monte Carlo settings
☒	☐	For hierarchical and complex designs, identification of the appropriate level for tests and full reporting of outcomes
☒	☐	Estimates of effect sizes (e.g. Cohen's *d*, Pearson's *r*), indicating how they were calculated

Our web collection on statistics for biologists contains articles on many of the points above.

Software and code

Policy information about availability of computer code

Data collection	Study data were collected and managed using REDCap (v9.1.8) electronic data capture tools hosted at Aarhus University. REDCap (Research Electronic Data Capture) is a secure, web-based software platform designed to support data capture for research studies, providing 1) an intuitive interface for validated data capture; 2) audit trails for tracking data manipulation and export procedures; 3) automated export procedures for seamless data downloads to common statistical packages; and 4) procedures for data integration and interoperability with external sources.
Data analysis	WES data was analyzed using the following software: bcl2fastq2 v2.17, Trim Galorel v04.1, bwa-mem v0.7.5, Picard suite v2.7.1, GATK v 3.7, VarScan2 v2.4.1, snpEff v4.3.i, bam-readcount v0.7.4, samtools suite v1.6.0, MuTect2 (GATK v3.7), Polysolver v1.0, Polyphen-2 and MutationAssessor v3.<br>RNA data was analyzed using the following software: Salmon v0.10.0, xCell (Web tool),<br>SNP data was analyzed using the following software: GenomeStudio v2.0.4<br>Quantification of protein markers was carried out using Visiopharm software v2018.9.5.5952<br>Output from the above was analyzed in R v3.6.1 and the following packages were used: SomaticSignatures v2.24.0, B5genome.Hsapiens.UCSC.hg19, MutationalPatterns v2.0.0,RTN v2.12.0, ASCAT v2.3, ConsensusClusteringPlus1.48.0, CHAMP v2.8.6, tximport v1.12.3, edgeR v3.26.8, consensusMIBC v.1.1, survminer 0.4.7, survival 3.1-12

For manuscripts utilizing custom algorithms or software that are central to the research but not yet described in published literature, software must be made available to editors and reviewers. We strongly encourage code deposition in a community repository (e.g. GitHub). See the Nature Research guidelines for submitting code & software for further information.

Data

Policy information about availability of data

All manuscripts must include a data availability statement. This statement should provide the following information, where applicable:

- Accession codes, unique identifiers, or web links for publicly available datasets
- A list of figures that have associated raw data
- A description of any restrictions on data availability

Raw sequencing and methylation data are deposited and available under controlled access at The European Genome-phenome Archive (EGA), which is hosted by the European Bioinformatics Institute (EBI) and the Centre for Genomic Regulation (CRG). Study accession numbers are: EGAS00001004507 (WES) [https://www.ebi.ac.uk/ega/studies/EGAS00001004507], EGAS00001004519 (SNP data) [https://www.ebi.ac.uk/ega/studies/EGAS00001004519], EGAS00001004505 (RNA-Seq) [https://www.ebi.ac.uk/ega/studies/EGAS00001004505], and EGAS00001004515 (EPIC BeadChip methylation data) [https://www.ebi.ac.uk/ega/studies/EGAS00001004515]. Normalized mRNA read counts are available in Supplementary Data 4. Source data are provided with this paper. TCGA WES, methylation and clinical data was accessed at [https://portal.gdc.cancer.gov/projects/TCGA-BLCA]. 450k methylation data for leukocytes were retrieved from the Gene Expression Omnibus (Series GSE32148) [https://www.ncbi.nlm.nih.gov/geo/query/acc.cgi?acc=GSE32148]. Only samples annotated as normal peripheral blood were used. The remaining data are available within the Article file, Supplementary Information or available from the authors upon request.

Field-specific reporting

Please select the one below that is the best fit for your research. If you are not sure, read the appropriate sections before making your selection.

- [x] Life sciences
- [ ] Behavioural & social sciences
- [ ] Ecological, evolutionary & environmental sciences

For a reference copy of the document with all sections, see nature.com/documents/nr-reporting-summary-flat.pdf

Life sciences study design

All studies must disclose on these points even when the disclosure is negative.

Sample size	Patients were included retrospectively, and no statistical methods were used to predetermine sample size. A total of 300 patients fulfilling the criteria necessary for a complete analysis were selected. These encompass diagnosis of muscle invasive bladder cancer followed by treatment according to national guidelines.
Data exclusions	Samples were excluded from an analysis platform (WES, RNA-seq, EPIC-aray or multiplex IHC) if a tumor sample was not suitable for the specific platform (low carcinoma cell fraction, low DNA or RNA concentration, fresh frozen material not available). This resulted in the partially overlapping multi-omics analyses.
Replication	Whole exome sequencing and RNA-Sequencing data obtained from tumor samples were not replicated. Furthermore, the staining of the tissue micro arrays with individual antibodies was only performed once for each tumor sample following optimization.
Randomization	Randomization was not relevant to our study as patients were included retrospectively. In this study we included patients with bladder cancer receiving chemotherapy, focusing in cisplatin-based chemotherapy. The study design did therefore not allow for randomly assign participants into a treatment group or a control group.
Blinding	Blinding was not relevant for this retrospective study. Patients were selected to represent all response groups (RECIST V.1; complete response, partial response, stable disease and progressive disease).

Reporting for specific materials, systems and methods

We require information from authors about some types of materials, experimental systems and methods used in many studies. Here, indicate whether each material, system or method listed is relevant to your study. If you are not sure if a list item applies to your research, read the appropriate section before selecting a response.

Materials & experimental systems

n/a	Involved in the study	
[ ]	[x]	Antibodies
[x]	[ ]	Eukaryotic cell lines
[x]	[ ]	Palaeontology and archaeology
[x]	[ ]	Animals and other organisms
[ ]	[x]	Human research participants
[ ]	[x]	Clinical data
[x]	[ ]	Dual use research of concern

Methods

n/a	Involved in the study	
[x]	[ ]	ChIP-seq
[x]	[ ]	Flow cytometry
[x]	[ ]	MRI-based neuroimaging

Antibodies

Antibodies used

Anti-CD8; clone:C8/144B; dilution: 1:150; incubation: 32 min; Dako, Agilent; cat#M710301-2; RRID:AB_2075537
Anti-CD3; clone:2GV6; dilution:Ready to use; incubation:24 min; Ventana Medical Systems, Inc.;cat#790-4341; RRID:NA
Anti-FOXP3; clone:SP97; dilution: 1:10; incubation:32 min;Thermo Fischer; cat#MA5-16365; RRID:AB_2537884
Pan Cytokeratin; clone:AE1/3; dilution: 1:100; incubation:16 min; Dako,Agilent; cat#GA005361-2; RRID:NA
Anti-CD163; clone:MRQ-26; dilution:Ready to use ; incubation:32 min; Ventana Medical Systems, Inc.; cat#;790-4341; RRID:NA
Anti-CD68 PG-M1; clone:PG-M1; dilution:1:100; incubation:32 min; Dako, Agilent; cat#GA61361-2; RRID:AB_2074844
Anti-CD20; clone:L26; dilution:Ready to use; incubation:32 min; Ventana Medical Systems, Inc.; cat#760-2531; RRID:NA
HLA-A,B,C; clone:EMRB-5; dilution:1:100; incubation:32 min; Abcam; cat#ab70328; RRID:AB_1269092
anti-mouse-HRP; Kit:OmniMap anti-Ms HRP (RUO), DISCOVERY; dilution: Ready to use; incubation:12 min; Ventana Medical Systems, Inc.; cat#760-4310
anti-rabit-HRP; Kit:OmniMap anti-Rb HRP (RUO), DISCOVERY; dilution: Ready to use; incubation:16 min; Ventana Medical Systems, Inc.;cat#760-4311
PD-L1;Clone:Sp263; dilution:Ready to use; incubation:60 min; Ventana Medical Systems, Inc.; Cat#790-4905; RRID:AB_2819099
PD-1;Clone:NAT105; dilution:Ready to use; incubation:32 min; Ventana Medical Systems, Inc.; Cat#760-4895; RRID:NA
Pan Cytokeratin; Clone:AE1/3; dilution:1:100; incubation:16 min; Dako, Agilent; Cat#GA005361-2; RRID:NA

Validation

Staining was performed at the Department of Pathology, Aarhus University Hospital on the Discovery ULTRA Staining instrument by a trained technician. Prior to multiplex staining each antibody was stained individually with a chromogenic detection method (DAB) to test specificity according to manufactures guidelines. Tonsil, spleen or lymph node tissue was used as a control tissue.

We have provided a link for the relevant data sheet for each antibody. The data sheet includes the manufacturer's validations statements, quality control procedures and relevant citations:
Anti-CD8; https://www.agilent.com/cs/library/packageinsert/public/108007002.PDF
Anti-CD3; https://pim-eservices.roche.com/eLD/api/downloads/49729da6-7333-ea11-fa90-005056a772fd?countryIsoCode=dk
Anti-FOXP3; https://www.thermofisher.com/order/genome-database/dataSheetPdf?producttype=antibody&productsubtype=antibody_primary&productId=MA5-16365&version=121
Pan Cytokeratin; https://www.agilent.com/cs/library/packageinsert/public/P02066DK_03.pdf or https://www.agilent.com/en/product/immunohistochemistry/antibodies-controls/primary-antibodies/cytokeratin-(dako-omnis)-76170#productdetails
Anti-CD163; https://www.e-labeling.eu/CMC44370030/61057/EN#remarkPopup
Anti-CD68 PG-M1; https://www.agilent.com/en/product/immunohistochemistry/antibodies-controls/primary-antibodies/cd68-(dako-omnis)-76227
Anti-CD20; https://pim-eservices.roche.com/eLD/api/downloads/50d4b69b-6833-ea11-fa90-005056a772fd?countryIsoCode=dk
HLA-A,B,C; https://www.abcam.com/hla-class-1-abc-antibody-emr8-5-ab70328.html
anti-mouse-HRP; https://pim-eservices.roche.com/eLD/api/downloads/2054601c-1513-ea11-fa90-005056a772fd?countryIsoCode=dk
anti-rabit-HRP; https://pim-eservices.roche.com/eLD/api/downloads/2054601c-1513-ea11-fa90-005056a772fd?countryIsoCode=dk
PD-L1;Clone: https://diagnostics.roche.com/global/en/products/tests/ventana-pd-l1-_sp263-assay1.html#productInfo
PD-1;Clone: https://pim-eservices.roche.com/eLD/web/dk/da/products/RTD001087?searchTerm=760-4895&catalog=HealthcareProfessional&orderBy=Relevance
Pan Cytokeratin; https://www.agilent.com/cs/library/packageinsert/public/107609005.PDF

Human research participants

Policy information about studies involving human research participants

Population characteristics

A total of 300 patients (Males = 235; Female = 65, mean±SD age at diagnosis 64 (±8)) with bladder receiving chemotherapy were included in the study; 62 received NAC before cystectomy (CX) and 245 received first-line chemotherapy upon detection of locally-advanced (T4b) or metastatic disease. Cisplatin-based chemotherapy was administered in ~98% of cases.

Recruitment

Patients were selected retrospectively based an bio bank availability. Therefore, patients were only included if they had sufficient bio-bank material for analysis at at least one of the analysis platforms.

Ethics oversight

National Committee an Health Research Ethics (NVK), Denmark. (#1706291)

Note that full information on the approval of the study protocol must also be provided in the manuscript.

Clinical data

Policy information about clinical studies

All manuscripts should comply with the ICMJEguidelines for publication of clinical research and a completedCONSORT checklist must be included with all submissions.

Clinical trial registration

Provide the trial registration number from ClinicalTrials.gov or an equivalent agency.

Study protocol

Note where the full trial protocol can be accessed OR if not available, explain why.

Data collection	*Describe the settings and locales of data collection, noting the time periods of recruitment and data collection.*
Outcomes	*Describe how you pre-defined primary and secondary outcome measures and how you assessed these measures.*
